# Evaluation of shape recovery algorithm for translucent objects

**DOI:** 10.1038/s41598-026-50949-6

**Published:** 2026-05-02

**Authors:** Takeaki Shimokawa

**Affiliations:** Graduate School of Information Sciences, Hiroshima City University, Hiroshima, 731-3194 Japan

**Keywords:** 3D shape, Orientation field, Specularity, Translucency, Vertical polarity, Mathematics and computing, Optics and photonics

## Abstract

The human visual system can estimate the three-dimensional shapes of translucent objects. However, the shape estimation of translucent objects is less accurate than that of opaque objects. The specular component is particularly important in the shape perception of translucent objects because it is robust, whereas the non-specular component is affected by translucency. Previously, we developed a shape recovery algorithm as a computational model of human shape perception from opaque specular images. The algorithm primarily uses the specular component and secondarily uses the non-specular component. In the current study, the shape recovery performance of this algorithm for translucent objects was evaluated against ground-truth shapes. The results showed that the reconstruction of the three-dimensional shapes of low-translucency objects by the algorithm was comparable to that of opaque specular images. A modification of the algorithm involving the reversal of the non-specular component was effective for high-translucency objects. However, even with this modification, the estimation accuracy for high-translucency objects was lower than that for opaque objects. These results provide new insight into the possible mechanisms of shape perception for translucent objects.

## Introduction

Humans can readily perceive a three-dimensional (3D) shape from a single two-dimensional (2D) image. Previous studies suggest that humans reconstruct 3D shapes not by inverting the image generation process from shape, material, and illumination, but by using image cues related to 3D shapes^1–4^. Local image orientation or direction, which is extracted in the primary visual cortex (V1)^5^, is one such image cue. For textured surfaces, image orientation corresponds to the orientation of the surface’s first derivative through texture foreshortening^6–8^. For specular surfaces, the image orientation corresponds to the orientation of the surface’s second derivative through specular reflection^1,9^. For matte surfaces, the direction of intensity gradients is related to the surface normal via lighting direction^2,3,10^.

**Fig. 1:**
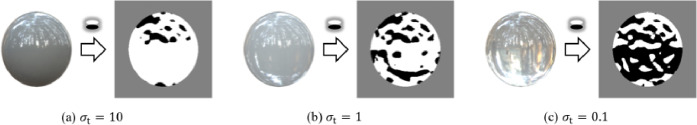
Relationship between translucency and vertical polarity of intensity gradient. Spheres are lit primarily from above by a natural illumination environment. Vertical polarity of each image, obtained by extracting a sign of V1-cell-like oriented filter response of vertical direction at each location, is depicted on the right. White represents positive and black represents negative. **a** Low translucency sphere (${\sigma_{\mathrm{t}}}={10^1}$). **b** Medium translucency sphere (${\sigma_{\mathrm{t}}}={10^0}$). **c** High translucency or transparent sphere (${\sigma_{\mathrm{t}}}={10^{ - 1}}$).

Although research into shape and material perception has traditionally focused on opaque object perception, the perception of translucent objects has been actively investigated in recent years^11–19^. Subsurface light transportation occurs in translucent objects instead of diffuse reflection, resulting in differences in non-specular components between translucent and opaque images. One notable difference in appearance is that, as translucency increases, the non-specular component is first blurred^11^ by subsurface scattering. Second, as translucency or transparency increases further, the non-specular component is contrast-reversed^11^ because light passing through a highly translucent or transparent object is less likely to be scattered and the object acts like a convex lens, which inverts the image upside down^20^. This affects both the material perception and the shape perception of translucent objects^13,17^. For example, Fig. 1 shows spheres of three levels of translucency generated using computer graphics under a natural illumination environment, in which the degree of translucency was controlled by setting different extinction coefficients^21^ inside the sphere: ${\sigma_{\mathrm{t}}}$ = 10, 1, 0.1 (see Methods for details). Because these spheres are convex and primarily lit from above, the intensity of the non-specular component of low translucency increases toward the top and decreases toward the bottom (see Fig. 1a). This vertical polarity of intensity gradient is extracted by oriented filter responses for the vertical direction. With increasing translucency, this relationship weakens (see Fig. 1b) then reverses (see Fig. 1c). In this way, translucency affects the relationship between image orientation or direction in the non-specular component and surface shape. In contrast, the specular component remains important for shape perception of translucent objects. Previous studies reported that the shape perception of translucent surfaces was weak in the absence of specular reflections^4,15^.

In our previous study, we developed a shape recovery algorithm to model human 3D shape perception from an opaque specular image^10^ (https://github.com/takeakishimokawa/ShapeFromSpecularity). The algorithm uses the orientation field, which is a collection of dominant orientations at every image location, and the vertical polarity of the intensity gradient as image cues to recover 3D shapes. As mentioned above, the orientation field of the specular component reveals the orientation of the surface’s second derivative. Furthermore, assuming that objects are illuminated from above, the vertical polarity reveals the sign of the surface’s second derivative (i.e., concave or convex) along the vertical direction. The vertical polarity is noisy because it is affected by the specular component (see the vertical polarity of the upper half of the sphere in Fig. 1a). Therefore, the algorithm uses the vertical polarity only for initial values of the signs of the surface’s second derivative and estimates these signs through optimization.

In the current study, the shape recovery algorithm was applied to translucent object images, and the shape recovery performance was evaluated. Because shape recovery from translucent images is inherently more difficult for both machines^22–24^ and humans than shape recovery from opaque images, this study focused on the evaluation of shape recovery performance against ground-truth shapes, which are more tractable than shapes perceived by humans. The purpose of this study was to build a computational model that recovers 3D shapes from image cues plausibly used by humans to obtain insight into human shape perception. The study addressed two main issues. First, the study was conducted to investigate the extent to which a shape recovery method developed for opaque specular images could be transferred to translucent images. Specular reflection plays an important role in the shape perception of translucent objects^4,15^, and the non-specular component is not yet contrast-reversed when the translucency is low. In such a case, it may be possible to recover the 3D shape from translucent object images using image cues and algorithms that are similar to those used for opaque specular images. Second, the study investigated how blurring or contrast-reversing of the non-specular component affects shape estimation when the translucency is high. If the non-specular component is only contrast-reversed, shape recovery may be achieved with the same accuracy as that for opaque specular images by using the reversed vertical polarity instead of the vertical polarity. The current study investigated whether and to what extent the use of the reversed vertical polarity is effective for 3D shape recovery from high translucency images.

## Methods

**Fig. 2:**
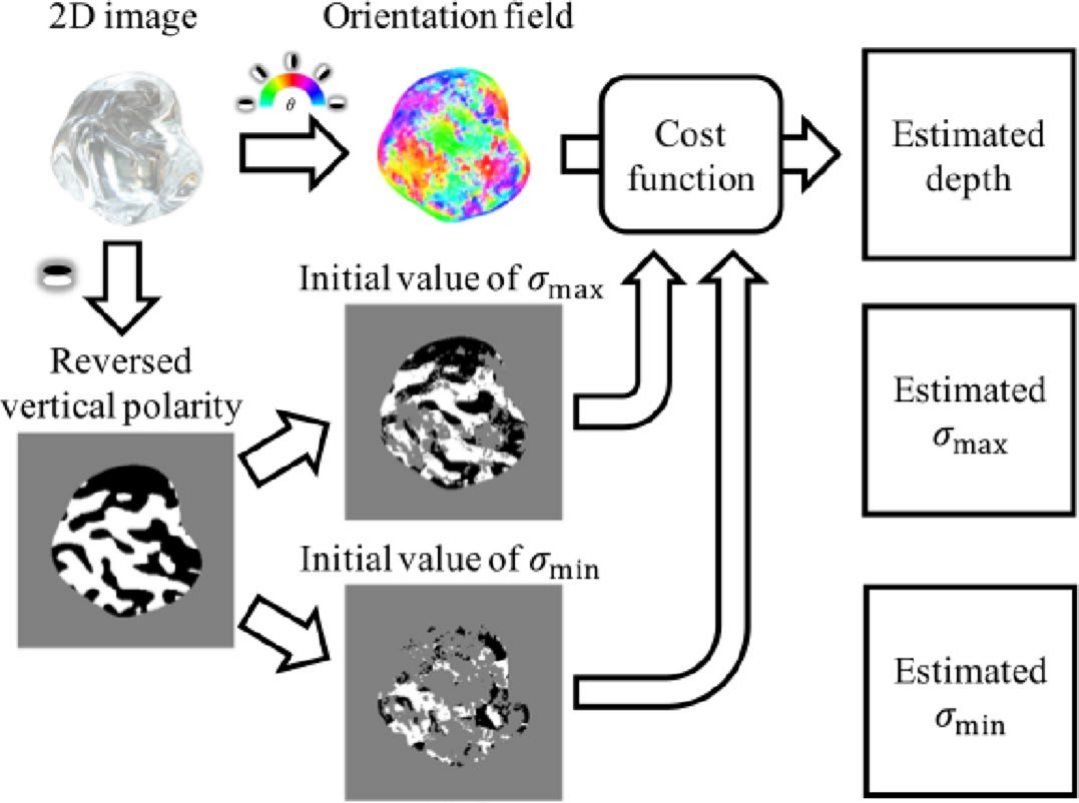
Flowchart of the shape recovery algorithm. The orientation field and the reversed vertical polarity are extracted from a translucent object image. The hue represents the image orientation that maximally stimulates the V1-cell-like oriented filter at each location. A cost function is formulated on the basis of the orientation field. Initial values of signs of surface second derivative, ${\sigma _{{\mathrm{max}}}}$ and ${\sigma _{{\mathrm{min}}}}$, are obtained by dividing the reversed vertical polarity. The estimated surface depth, ${\sigma _{{\mathrm{max}}}}$, and ${\sigma _{{\mathrm{min}}}}$ are obtained by minimizing the cost function.

Figure 2 shows the flowchart of the algorithm to recover 3D surface depth from a single translucent or transparent object image. The main procedure is as follows. First, the orientation field is extracted from an image; second, the cost function is formulated on the basis of the orientation field; finally, the 3D shape is recovered by minimizing the cost function. Additionally, the vertical polarity is extracted from the image to resolve concave/convex ambiguity. Although the vertical polarity is used for an opaque specular image^10^, the reversed vertical polarity is used for a high translucency image. The initial values of the surface second derivative signs, ${\sigma _{{\mathrm{max}}}}$ and ${\sigma _{{\mathrm{min}}}}$, are calculated on the basis of the vertical polarity and used to minimize the cost function.

As a precondition to 3D shape recovery, it is assumed that the image region in which the object exists is known. The object region is denoted as ${{\boldsymbol{\Omega}}}$. The resolution of the 3D shape recovery is $256 \times 256$ pixels. A Cartesian coordinate is set on the image plane, where the *x*- and *y*-axes represent the horizontal and vertical axes of an image plane, respectively, and the *z* axis represents the direction toward the viewer. The depth of the 3D object surface is represented as $z\left( {x,y} \right)$.

### Images and extraction of image cues

The images of 12 different 3D shapes were used to evaluate the shape recovery algorithm. The images had a resolution of $1024 \times 1024$ pixels. These images were rendered using Mitsuba 3 renderer software^21^. The albedo of the medium was set to 0.75. The extinction coefficient ${\sigma_{\mathrm{t}}}$ of the medium inside the object was set to ${10^{ - 1}}$, ${10^{ - 0.5}}$, ${10^0}$, ${10^{0.5}}$, and ${10^1}$ to generate images with various degrees of translucency. A smooth dielectric material was used with a refractive index value of 1.49 (acrylic glass). The number of samplers per pixel was set to ${2^{20}}$. The 3D shapes of objects #1–6 were randomly generated using spherical harmonics with increasing complexity. The 3D shapes of objects #7–12 were human-made. These 3D shapes were used in our previous studies^8,10,25,26^. For the natural illumination environment, a high dynamic range image from the Devebec dataset was used (https://www.pauldebevec.com/Probes/; Eucalyptus Grove). Note that the angle of the illumination environment around the *y*-axis differed by 20° between for objects #1–6 and objects #7–12. These conditions were the same as those reported in our previous study^10^.

The orientation field was extracted as follows. The image orientation $\theta (x,y)$ is the angle that maximizes the magnitude of the response *p* of the oriented filter: $\theta (x,y)={\mathrm{argma}}{{\mathrm{x}}_{{\theta ^\prime }}}{p^2}({\theta ^\prime }(x,y))$. Image anisotropy $\alpha (x,y)$ is defined by the ratio of the minimum and maximum magnitudes of the response with respect to its angle^1^ as $\alpha (x,y)=1 - \sqrt {\frac{{p_{{{\mathrm{min}}}}^{2}(x,y)}}{{p_{{{\mathrm{max}}}}^{2}(x,y)}}}$. The steerable pyramid^27,28^ was used to extract the image orientation in the same manner reported in our previous study^10^. Although orientation responses at the finest spatial scale were extracted in our previous study, those at the second-finest spatial scale ($512 \times 512$ pixel resolution) were extracted in this study because of their relative robustness to the rendering noise of translucent objects, except for object #9. Those at the finest spatial scale were extracted for object #9 because of its small object region.

The vertical polarity of intensity gradient ${p_{\mathrm{v}}}(x,y)$ was obtained by extracting the sign of the oriented filter response of the vertical direction ($\theta =0^\circ$) as ${p_{\mathrm{v}}}(x,y)={\mathrm{sgn(}}{p_{\theta =0^\circ }}(x,y){\mathrm{)}}$. The reversed vertical polarity was given by $- {p_{\mathrm{v}}}(x,y)={\mathrm{sgn}}({p_{\theta =180^\circ }}(x,y))$. The steerable pyramid was used to extract the vertical polarity in the same manner reported in our previous study^10^.

The signs of the apparent curvature of the image contour were derived in the same manner reported in our previous study (see Supplementary Fig. 5 in our previous study^10^.

The image cues (i.e., orientation field and vertical polarity) were evaluated as follows. The orientation field error was quantified by the mean absolute errors throughout the object region between the image and surface orientations and between the image and surface anisotropies. The vertical polarity was evaluated by calculating the correct ratio between the vertical polarity, $\:{p}_{\mathrm{v}}$, and the surface derivative sign along the $\:y$-axis, ${\sigma_{y}}$. 

### Curvature formulation and cost function

The surface shape of objects is described using the Hessian matrix $H\left( z \right)$ of surface depth $z\left( {x,y} \right)$^10^. $H\left( z \right)$ is diagonalized with rotation matrix *R* as

1 $$H\left( z \right)=\left( {\begin{array}{*{20}{c}} {\frac{{{\partial ^2}z}}{{\partial {x^2}}}}&{\frac{{{\partial ^2}z}}{{\partial x\partial y}}} \\ {\frac{{{\partial ^2}z}}{{\partial x\partial y}}}&{\frac{{{\partial ^2}z}}{{\partial {y^2}}}} \end{array}} \right)= - R\left( {{\theta _{\mathrm{s}}}} \right)\left( {\begin{array}{*{20}{c}} {{k_{{\mathrm{min}}}}}&0 \\ 0&{{k_{{\mathrm{max}}}}} \end{array}} \right)R\left( { - {\theta _{\mathrm{s}}}} \right),$$

where ${k_{{\mathrm{max}}}}$ and ${k_{{\mathrm{min}}}}$ are the eigenvalues of the larger and smaller magnitudes. ${\theta _{\mathrm{s}}}$, which indicates the angle of the small surface second derivative, is called the surface orientation. Surface anisotropy ${\alpha _{\mathrm{s}}}$ is defined^1^ as ${\alpha _{\mathrm{s}}}=1 - \sqrt {k_{{{\mathrm{min}}}}^{2}/k_{{{\mathrm{max}}}}^{2}}$. The signs of the large and small surface second derivatives are represented as ${\sigma _{{\mathrm{max}}}} \in \left\{ {+1, - 1} \right\}$ and ${\sigma _{{\mathrm{min}}}} \in \left\{ {+1, - 1} \right\}$, respectively. $+1$ and $- 1$ correspond to convex and concave shapes. The magnitude of the large surface second derivative is represented as ${k_{\mathrm{a}}}=\left| {{k_{{\mathrm{max}}}}} \right|$. Using these variables, the surface second derivatives are described:

2 $${k_{{\mathrm{max}}}}={k_{\mathrm{a}}}{\sigma _{{\mathrm{max}}}},$$

3 $${k_{{\mathrm{min}}}}=\left( {1 - {\alpha _{\mathrm{s}}}} \right){k_{\mathrm{a}}}{\sigma _{{\mathrm{min}}}}.$$

The cost function consists of two terms^10^: the second derivative constraint given by orientation field *C* and boundary condition *B*:

4 $$E=C+B.$$

The second derivative constraint is derived on the basis of the relationship^1,9^ that the image orientation approximates surface orientation $\theta \approx {\theta _{\mathrm{s}}}$ and the image anisotropy approximates surface anisotropy $\alpha \approx {\alpha _{\mathrm{s}}}$. *C* is defined as the sum of the squared differences of the surface second derivative from the constraint given by the orientation field. Boundary condition *B* consists of the following three terms: ${B_0}+{B_1}+{B_c}$. ${B_0}$ is introduced to resolve the translation ambiguity along the depth direction. ${B_1}$ is introduced to resolve the ambiguity of the affine transformation^29^. ${B_c}$ incorporates the knowledge that, near the boundary, ${\sigma _{{\mathrm{max}}}}=+1$ and ${\sigma _{{\mathrm{min}}}}$ equals the sign of the apparent curvature of the 2D contour^30^, assuming that the 3D surface near the boundary is smooth and differentiable. The resultant cost function *E* is a function of ${\sigma _{{\mathrm{max}}}}$, ${\sigma _{{\mathrm{min}}}}$, and ${k_{\mathrm{a}}}$. The initial values of ${\sigma _{{\mathrm{max}}}}$ and ${\sigma _{{\mathrm{min}}}}$ obtained by the vertical polarity are used for optimization. The details of the derivation and minimization of the cost function were reported in our previous study^10^.

### Relationship between vertical polarity and surface second derivative signs

With the prior knowledge that the object is illuminated from above, it is possible to derive the relationships among the vertical polarity, ${p_{\mathrm{v}}}$, and the surface second derivative sign along the *y*-axis, ${\sigma _y}={\mathrm{sgn}}\left( { - \frac{{{\partial ^2}z}}{{\partial {y^2}}}} \right)$. If an opaque object is illuminated from above, the surface luminance tends to be higher as the surface slant $- \frac{{\partial z}}{{\partial y}}$ is increased^10^. By taking a derivative of this relationship with respect to *y* and taking the sign, the following approximation is obtained:

5 $${p_{\mathrm{v}}} \approx {\sigma _y}.$$

However, it has been reported that this relationship is reversed for objects with high translucency^11^ as

6 $$- {p_{\mathrm{v}}} \approx {\sigma _y}.$$

The surface derivative sign along the *y*-axis can be described using Eqs. (1), (2), and (3) as ${\sigma _y}={\mathrm{sgn}}\left( {{\sigma _{{\mathrm{max}}}}{{\cos }^2}{\theta _s}+{\sigma _{{\mathrm{min}}}}\left( {1 - {\alpha _s}} \right){{\sin }^2}{\theta _s}} \right)$. The approximation of orientation $\theta \approx {\theta _{\mathrm{s}}}$ and anisotropy $\alpha \approx {\alpha _{\mathrm{s}}}$ is then used:

7 $${\sigma _y} \approx {\mathrm{sgn}}\left( {{\sigma _{{\mathrm{max}}}}{{\cos }^2}\theta +{\sigma _{{\mathrm{min}}}}\left( {1 - \alpha } \right){{\sin }^2}\theta } \right).$$

Object region ${{\boldsymbol{\Omega}}}$ is divided into two regions: ${\cos ^2}\theta \geqslant \left( {1 - \alpha } \right){\sin ^2}\theta$ holds in ${{{\boldsymbol{\Omega}}}_{\mathrm{a}}}$, but not in ${{{\boldsymbol{\Omega}}}_{\mathrm{b}}}$. The following relationship is then obtained:

8 $${\sigma _y}\left( {x,y} \right) \approx \left\{ {\begin{array}{*{20}{c}} {{\sigma _{{\mathrm{max}}}}\left( {x,y} \right)}&{\left( {x,y \in {{{\boldsymbol{\Omega}}}_{\mathrm{a}}}} \right)} \\ {{\sigma _{{\mathrm{min}}}}\left( {x,y} \right)}&{\left( {x,y \in {{{\boldsymbol{\Omega}}}_{\mathrm{b}}}} \right)} \end{array}} \right..$$

By combining Eqs. (8) and (5) or (6), the initial values of $\:{\sigma\:}_{\mathrm{max}}$ and $\:{\sigma\:}_{\mathrm{min}}$ for optimization are obtained from the vertical polarity.

### Evaluation of recovered depth

**Fig. 3:**
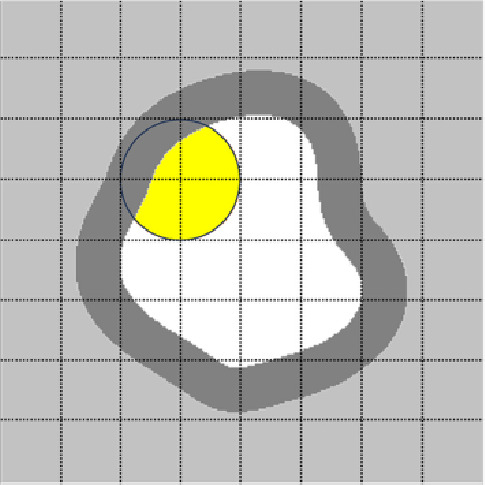
One of the areas for calculating local interior depth correlation in object #1. In this example, the circle is centered at the third intersection from the top and the third from the left. In the calculation, the areas outside the object region, depicted in light gray, and the areas close to the boundary, depicted in dark gray, are excluded. The correlation between the recovered depth and the true depth is calculated in the area depicted in yellow, where the circle overlaps with the interior of the object, excluding the dark gray area near the boundary. If the yellow area is smaller than half the area of the circle, which is not the case in this example, no correlation is calculated. This process is repeated for circles centered at all intersections, and the average of the obtained correlation coefficients is the local interior depth correlation.

The shape recovery performance was quantified in the same manner reported in our previous study^10^. Two depth correlations were used: global and local interior. The global depth correlation is the correlation coefficient of the recovered and true depths throughout the object region. However, the global depth correlation tends to become high as long as the depth around the boundary is small, because the true depth is generally very small around the boundary and modest inside the object region. In other words, the global depth correlation is sensitive to the depth around the boundary and insensitive to the details of the shape within the object region. Therefore, the local interior depth correlation is also calculated for evaluation as follows. First, a grid is set to divide the vertical and horizontal axes of the image into eight regions, respectively (at 32-pixel intervals, dotted line in Fig. 3). Second, a circle is centered at an intersection of the grid with a radius of 32 pixels. Third, a depth correlation is measured in the intersection of the circle and the object area (yellow area in Fig. 3) after removing the area near the boundary (within 24 pixels from the boundary, dark gray area in Fig. 3). A depth correlation is not measured if the intersection area is smaller than half of the circle’s area. Fourth, the depth correlation values are averaged. The local interior depth correlation is not affected by the shapes near the boundary and is sensitive to the agreement of the concavity and the convexity inside the object region. Note that the local interior depth correlation was not evaluated for objects #9 and #11 because most of the object region was near the boundary.

## Results

**Fig. 4:**
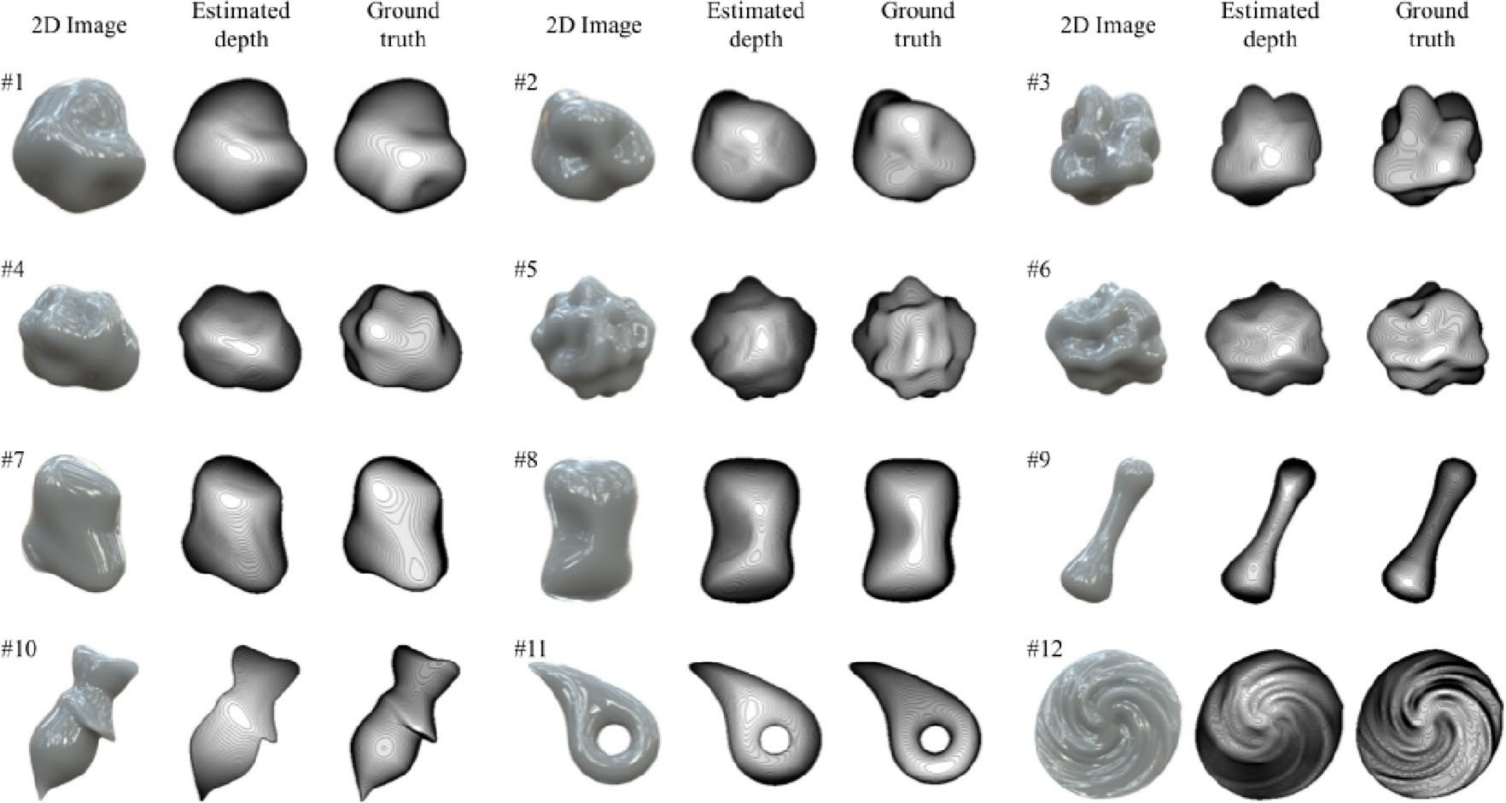
Recovered 3D shapes from low translucency object images (${\sigma_{\mathrm{t}}}={10^1}$). The recovered surface depths and the ground-truth surface depths are represented by depth maps with superimposed contour lines. The vertical polarity was used for all 12 objects for the initial values of the surface second derivative signs.

**Fig. 5:**
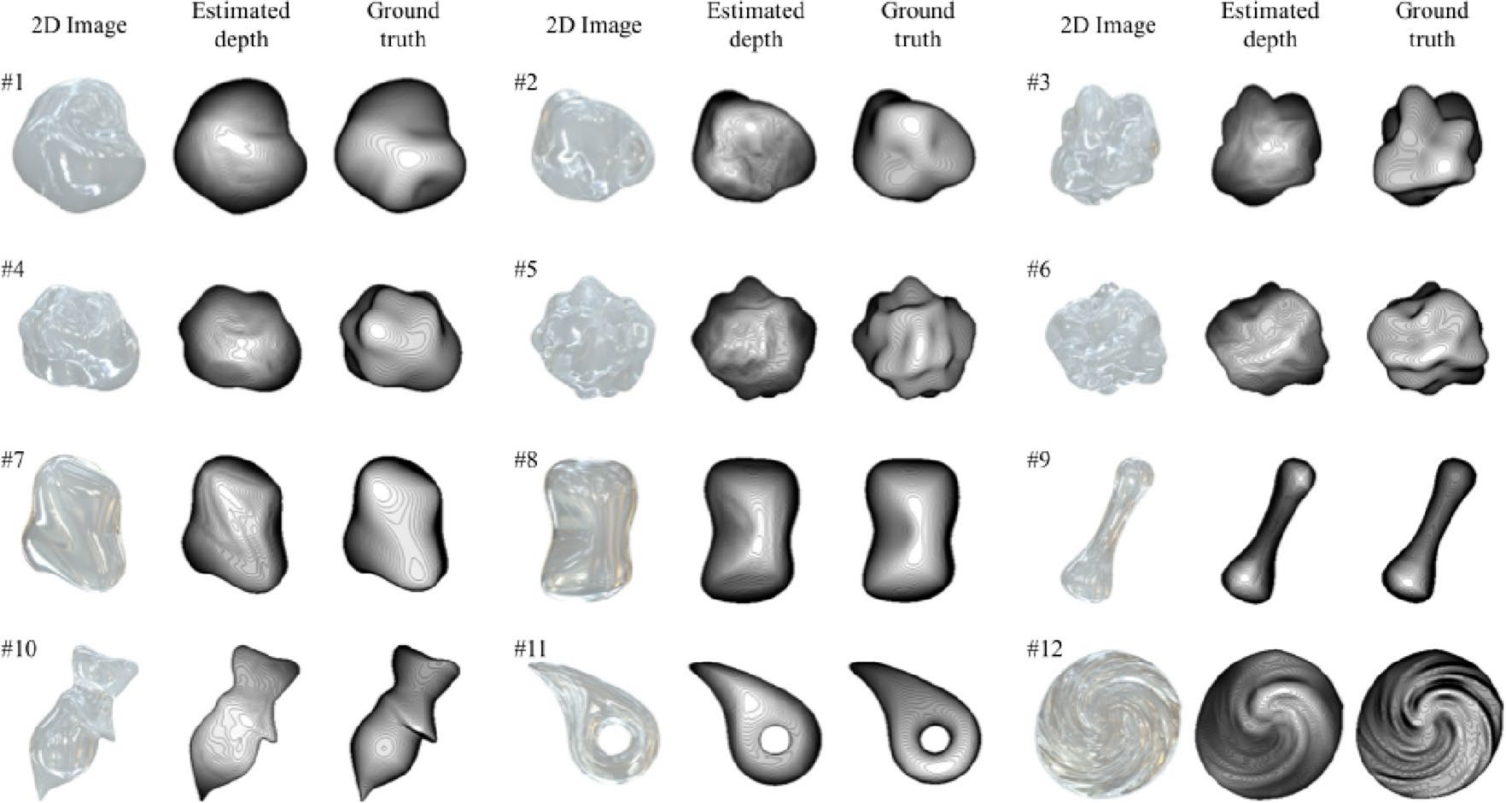
Recovered 3D shapes from medium translucency object images (${\sigma_{\mathrm{t}}}={10}^{0}$). The recovered surface depths and the ground-truth surface depths are represented by depth maps with superimposed contour lines. The vertical polarity was used for objects #1, #2, #3, #4, #5, #6, and # 12, and the reversed vertical polarity was used for objects #7, #8, #9, #10, and #11 for the initial values of the surface second derivative signs.

**Fig. 6:**
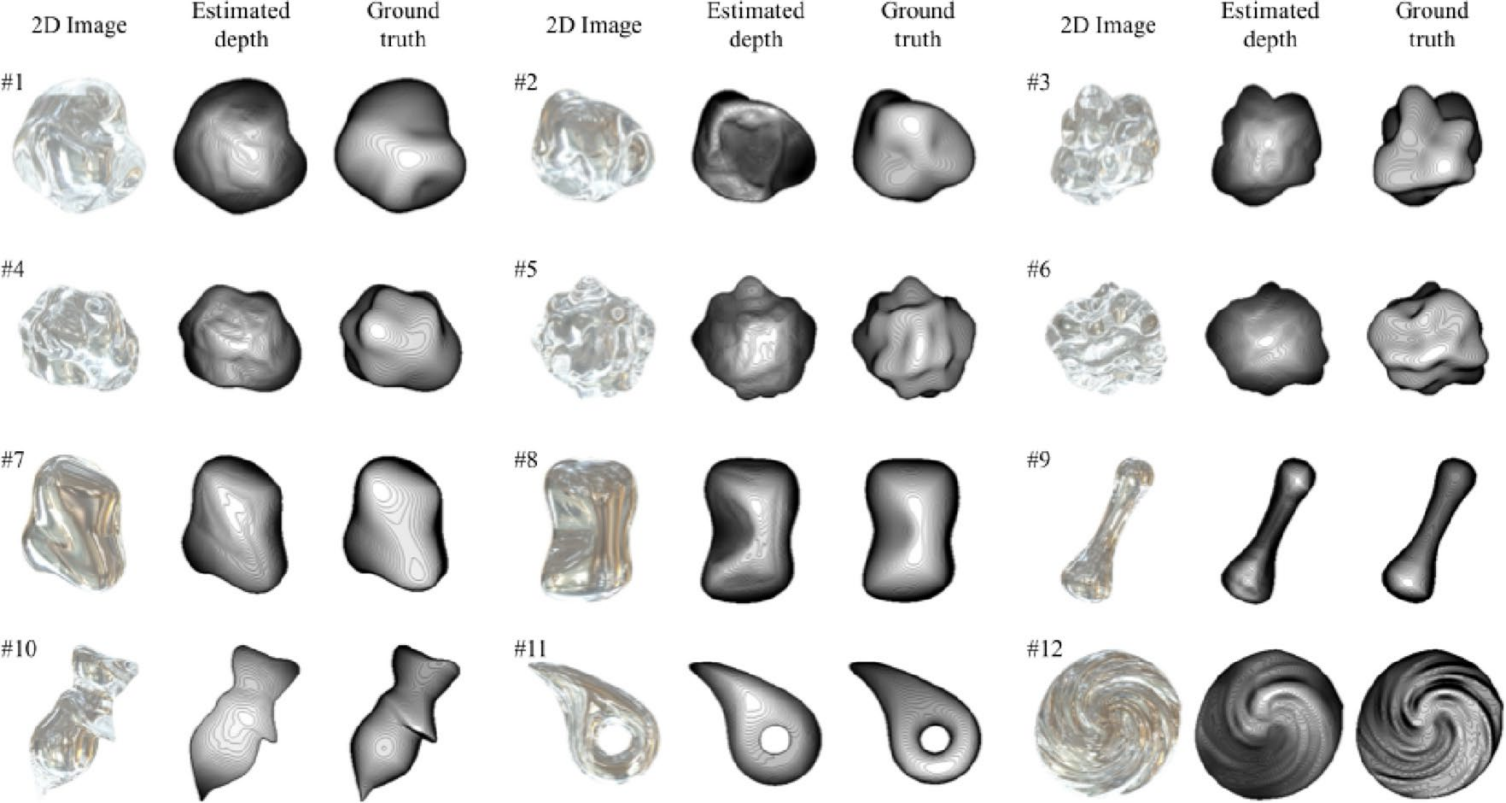
Recovered 3D shapes from high translucency or transparent object images (${\sigma_{\mathrm{t}}}={10}^{-1}$). The recovered surface depths and the ground-truth surface depths are represented by depth maps with superimposed contour lines. The vertical polarity was used for objects #2, #9, and # 12, and the reversed vertical polarity was used for objects #1, #3, #4, #5, #6, #7, #8, #10, and #11 for the initial values of the surface second derivative signs.

To evaluate the shape recovery algorithm, 12 computer-generated 3D objects with various degrees of translucency were used (${\sigma_{\mathrm{t}}}$ = ${10^{ - 1}},{10^{ - 0.5}},{10^0},{10^{0.5}},{10^1}$) (see 2D images of Fig. 4 for ${\sigma_{\mathrm{t}}}$ = ${10^1}$, Fig. 5 for ${\sigma_{\mathrm{t}}}$ = ${10^0}$, and Fig. 6 for ${\sigma_{\mathrm{t}}}$ = ${10^{ - 1}}$). Examples of recovered shapes from the translucent object images with the ground-truth shapes are shown in Figs. 4, 5 and 6. The depths are represented in grayscale; nearer surfaces are lighter, and distant surfaces are darker. Additionally, 50 contour lines are superimposed to aid in the depiction. Note that Figs. 4, 5 and 6 show better results using either the vertical polarity or the reversed vertical polarity for optimization.

Table 1 summarizes the errors of the image cues that were averaged across the 12 objects. The mean absolute errors of the orientation and anisotropy in the case of opaque glossy objects with the same shapes in our previous study were 11.3$\:^\circ\:$ and 0.15, respectively^10^. The orientation field errors increased with increasing translucency. The correct ratio of the vertical polarity in the case of opaque glossy objects in our previous study^10^ was 0.79. The errors of the vertical polarity also increased with increasing translucency. In the case of high translucency (${\sigma_{\mathrm{t}}}$ = $\:{10}^{-0.5},{10}^{-1}$), the correct ratio was below 0.5. In this case, the correct ratio of the reversed vertical polarity exceeded that of the vertical polarity because the summation of the correct ratio of the vertical polarity and that of the reversed vertical polarity is 1. This indicates that the vertical polarity and the reversed vertical polarity are effective for shape recovery from objects with low and high translucency, respectively.

**Table 1:** Evaluation of image cues averaged across the 12 objects.

Extinction coefficient	Mean absolute error		Correct ratio
$\:{\sigma\:}_{\mathrm{t}}$	Orientation	Anisotropy	Vertical polarity
10^1^	14.7°	0.16	0.79
10^0.5^	16.1°	0.17	0.68
10^0^	18.4°	0.17	0.49
10^–0.5^	21.4°	0.19	0.43
10^–1^	22.7°	0.20	0.43

**Fig. 7:**
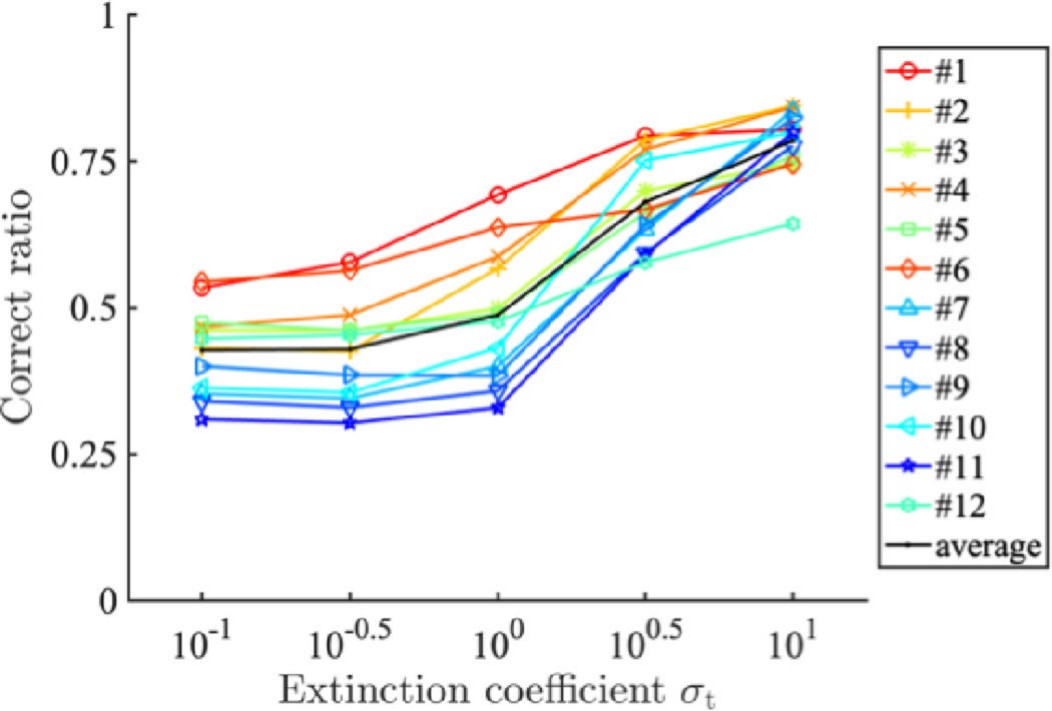
Correct ratio of vertical polarity of various degrees of translucency for each object. Each object is indicated by a combination of color and symbol. The black line indicates the averaged value across the 12 objects.

Figure 7 shows the correct ratio of vertical polarity for each object. The color indicating each object was determined by the correct ratio value in the medium translucency (${\sigma_{\mathrm{t}}}$ = 10^0^). There was a tendency for the correct ratio of objects indicated by reddish colors to be higher than that of objects indicated by bluish colors, regardless of translucency. The results indicated that whether the vertical polarity or the reversed vertical polarity was effective for shape recovery depended not only on its translucency but also its shape.

**Fig. 8:**
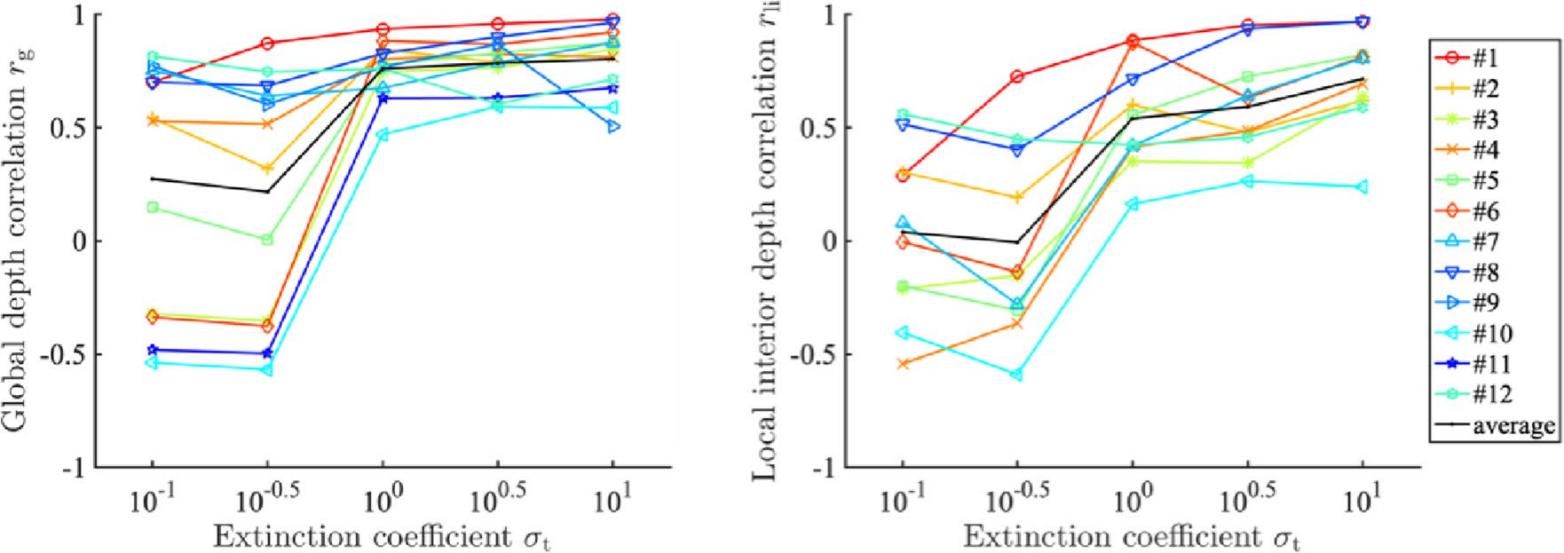
Shape recovery performance of various degrees of translucency when the vertical polarity was used for the initial values for optimization. Each object is indicated by a combination of color and symbol. The black line indicates the averaged value across the 12 objects.

**Fig. 9:**
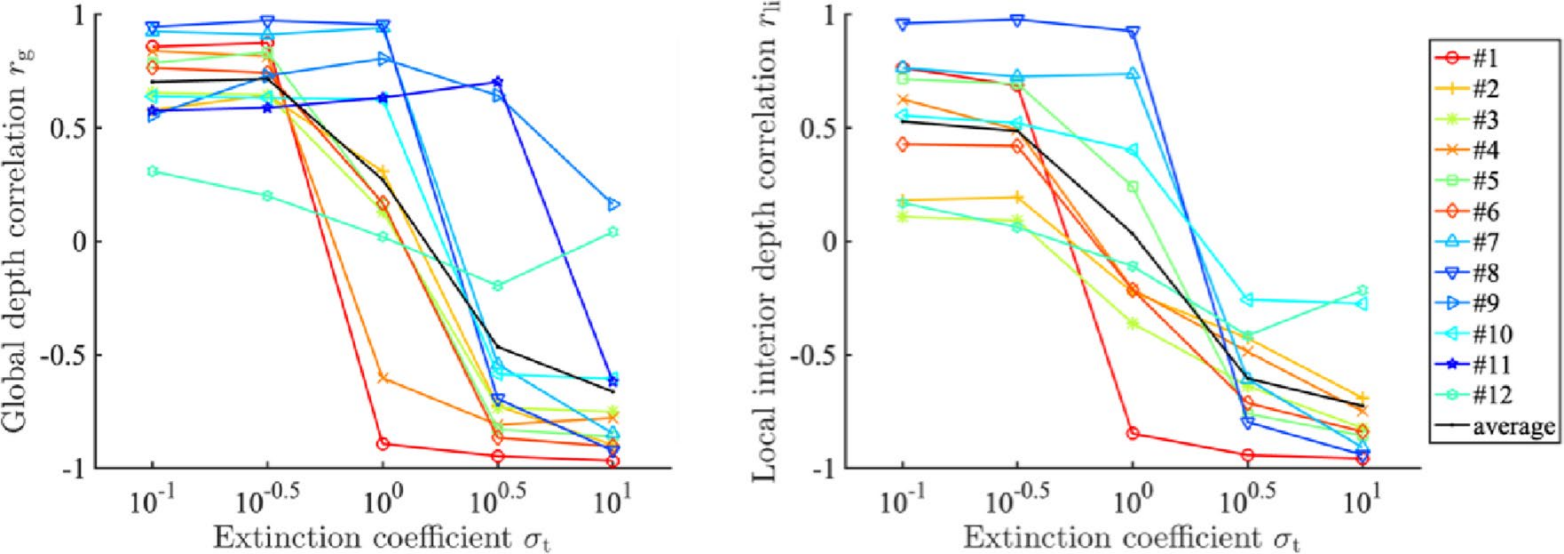
Shape recovery performance of various degrees of translucency when the reversed vertical polarity was used for the initial values for optimization. Each object is indicated by a combination of color and symbol. The black line indicates the averaged value across the 12 objects.

Figure 8 shows the shape recovery performance when the vertical polarity was used for optimization. Although the local interior depth correlations were lower than the global depth correlations, both correlations exhibited the same tendency. The depth correlations were high for low-translucency objects and tended to be high in objects indicated by reddish colors. Figure 9 shows the shape recovery performance when the reversed vertical polarity was used for optimization. Both depth correlations exhibited the same tendency. The depth correlations were high for high-translucency objects and tended to be high in objects indicated by bluish colors.

For the low translucency object images (${\sigma_{\mathrm{t}}}$ = 10^1^) shown in Fig. 4, the shape recovery performance of all 12 objects was higher when the vertical polarity was used for optimization. In this best choice, the average values of the global depth correlation $\:{r}_{\mathrm{g}}$ and the local interior depth correlation $\:{r}_{\mathrm{l}\mathrm{i}}$ across the 12 objects were 0.80 and 0.72, respectively. This shape recovery performance was slightly worse than that for opaque glossy objects in the previous study^10^ ($\:{r}_{\mathrm{g}}=0.85$ and $\:{r}_{\mathrm{l}\mathrm{i}}=0.76$). The shape recovery appeared to be successful for all objects except object #10, for which the local interior depth correlation $\:{r}_{\mathrm{l}\mathrm{i}}$ was 0.24 (see Fig. 8).

For the medium translucency object images (${\sigma_{\mathrm{t}}}$ = $\:{10}^{0}$) shown in Fig. 5, the shape recovery performance for #1, #2, #3, #4, #5, #6, and #12 was higher when the vertical polarity was used, and the shape recovery performance for #7, #8, #9, #10, and #11 was higher when the reversed vertical polarity was used for optimization. In this best choice, the average values of the global depth correlation $\:{r}_{\mathrm{g}}$ and the local interior depth correlation $\:{r}_{\mathrm{li}}$ across the 12 objects were 0.81 and 0.62, respectively. Although the overall performance was worse than that for low translucency object images, the shape recovery was still successful for objects #1 ($\:{r}_{\mathrm{li}}=0.88$), #6 ($\:{r}_{\mathrm{li}}=0.88$), and #8 ($\:{r}_{\mathrm{li}}=0.93$).

For the high translucency or transparent object images (${\sigma_{\mathrm{t}}}$ = 10^–1^) shown in Fig. 6, the shape recovery performance of objects #2, #9, and # 12 was higher when the vertical polarity was used for optimization. The shape recovery performance of objects #1, #3, #4, #5, #6, #7, #8, #10, and # 11 was higher when the reversed vertical polarity was used for optimization. In this best choice, the average values of the global depth correlation $\:{r}_{\mathrm{g}}$ and the local interior depth correlation $\:{r}_{\mathrm{li}}$ values across the 12 objects were 0.76 and 0.58, respectively. The overall performance was much worse than that for the low translucency object images. The shape recovery failed for objects #2 ($\:{r}_{\mathrm{li}}=0.30$) and #3 ($\:{r}_{\mathrm{li}}=0.11$). The shape recovery was still successful for object #8 ($\:{r}_{\mathrm{li}}=0.96$).

## Discussion

A computational model for shape recovery from image cues plausibly used by humans was evaluated for translucent objects against ground-truth shapes. The results revealed that a 3D shape could be reconstructed from a single translucent object image using the model, although the estimation accuracy was lower when the object was highly translucent or transparent. It is difficult to model the light passing through a translucent object. To the best of the author’s knowledge, no shape recovery algorithms have previously been developed for a single translucent object image^19^. The current results showed that a shape recovery algorithm developed for opaque glossy objects on the basis of the orientation field and vertical polarity was directly applicable to low-translucency objects. Additionally, the results showed that it was necessary to use the reversed vertical polarity instead of the vertical polarity for high translucency or transparent objects because the relationship between the non-specular component and the 3D shape was reversed for high-translucency objects^11^. These results suggest that similar mechanisms could be used for opaque specular and low-translucency objects, whereas a modified mechanism might be used for high-translucency objects in shape recognition processing in human vision. However, because human shape perception was not investigated in the current study, psychophysical experiments will be required to verify these implications in future research.

The orientation field error for low-translucency objects was comparable to that for opaque glossy objects^10^ (see Table 1). The shape recovery algorithm tested in the current study assumed the specular reflection on the object’s surface and used the orientation field of the specular component to obtain the surface second derivative constraint. Because the subsurface light transport process makes light blurred before re-emerging from the surface in highly scattering media^31–33^, the extraction of the orientation field would be expected to be minimally affected by subsurface scattering. However, as the scattering coefficient decreases, the light passing through an object is not blurred and produces different image orientations from those caused by the specular component, leading to a difference between the image orientation and the surface orientation. As a result, the estimation accuracy decreased for highly translucent or transparent objects.

With increasing translucency, the correct ratio of the vertical polarity not only reversed, but also approached 0.5 (see Table 1). Previous studies reported that the relationship between the surface luminance and the surface orientation is also affected by other factors, such as the surface shape with increasing translucency^15,19^. Thus, the light passing through a translucent object does not perfectly obey either Eq. (5) or Eq. (6). This phenomenon may also account for the decreased estimation accuracy observed for highly translucent objects, even when the reversed vertical polarity was used.

The shape recovery algorithm for translucent objects tested in the current study involved several limitations that should be considered. First, the algorithm requires the presence of the specular component in the image. Similarly, human shape perception has been reported to become extremely weak in the absence of specular reflection of translucent objects^4,15^. Second, the current algorithm does not have the ability to automatically determine whether the vertical polarity or the reversed vertical polarity should be used. Although the degree of translucency, the shape, and, likely, the illumination environment affect the correct ratio of the vertical polarity (see Fig. 7), they are not obtained from a single image. Only the 3D surface curvature sign near the boundary is directly given by the sign of the apparent curvature of the 2D contour^30^. Therefore, the right-hand side of Eq. (5) or Eq. (6) (i.e., ${\sigma_{y}}$) is known near the boundary, and it might be possible to determine whether the given image follows Eq. (5) or Eq. (6) by comparing $\:{p}_{\mathrm{v}}$ and ${\sigma_{y}}$ near the boundary.

The current findings suggest two possible directions for future research. First, future studies could incorporate further findings from psychophysical studies of image cues that humans use for shape perception from translucent objects. For example, the intensity of light returned from an object tends to be high in the convex region and low in the concave region in translucent objects^15,19^. Adding these cues as a constraint for ${\sigma _{{\mathrm{max}}}}$, ${\sigma _{{\mathrm{min}}}}$, and $\:{k}_{\mathrm{a}}$ would be expected to improve the estimation accuracy of the algorithm. Second, it may be valuable for future research to investigate the relationship between the recovered shape and human shape perception. Because the shape recovery algorithm tested in the present study was developed on the basis of psychophysical findings, the recovered shapes may have some resemblance to perceived shapes. For example, the recovered shapes of objects #1, #4, and #5 deviated from ground-truth shapes (see Fig. 6). The shapes perceived by humans would also be expected to deviate from ground-truth shapes. Identifying the similarities and dissimilarities of the two deviations may be helpful for better understanding human shape perception and improving the shape recovery algorithm.

## Conclusion

The performance of a shape recovery algorithm using the orientation field and the vertical polarity was evaluated for translucent objects. The estimation accuracy was high for low-translucency objects. This suggests that the same shape recovery algorithm could be applicable for opaque glossy objects and low-translucency objects if these image cues are used for shape reconstruction. However, it was necessary to use the reversed vertical polarity instead of the vertical polarity for high-translucency objects, and the estimation accuracy was lower than that for opaque and low-translucency objects, despite this modification.

## Data Availability

The image dataset generated and analyzed during the current study is available at https://doi.org/10.5281/zenodo.18830497. The code for the shape recovery algorithm is available at https://doi.org/10.5281/zenodo.18829112.
